# SMASH standardised perioperative management of patients operated with acute abdominal surgery in a high-risk setting

**DOI:** 10.1186/s13104-020-05030-4

**Published:** 2020-03-31

**Authors:** Terje Jansson Timan, Ninni Sernert, Ove Karlsson, Mattias Prytz

**Affiliations:** 1 Institute of Clinical Sciences, Department of Surgery, Sahlgrenska Academy, University of Gothenburg, Gothenburg, Sweden; 2 Department of Research and Development, NU-Hospital group, Trollhättan, Sweden; 3 Department of Anaesthesiology and Intensive Care Unit, NU-Hospital group, Trollhättan, Sweden; 4 Institute of Clinical Sciences, Department of Orthopaedics, Sahlgrenska Academy, University of Gothenburg, Gothenburg, Sweden; 5 Institute of Clinical Sciences, Department of Anaesthesiology & Intensive care, Sahlgrenska Academy, University of Gothenburg, Gothenburg, Sweden; 6 Department of Surgery, NU-Hospital Group, Trollhättan, Sweden

**Keywords:** Surgery, Anaesthesiology, Emergency, Laparotomy, Abdominal, Critically ill, Perioperative, Management, Mortality

## Abstract

**Objective of the study:**

Emergency laparotomy and other high-risk acute abdominal surgery procedures have a high mortality rate. The perioperative management of these patients is complex and poses several challenges. The objective of the study is to implement and evaluate the outcome of protocol-based standardised care for patients in need of acute abdominal surgery in a Swedish setting. NÄL is a large county hospital in Sweden serving a population of approximately 270,000 inhabitants. The study seeks to determine whether standardised protocol-based perioperative management in emergency abdominal surgical procedures leads to a better outcome measured as short- and long-term mortality and postoperative complications compared with the present standard in Swedish routine care. The study is ongoing, and this article describes the methodology used in the study and discusses the benefits and limitations the study design.

**Results:**

There are no results so far. The inclusion rate for the first 22 months is as expected; 404 patients have been included and protocols have been followed and reviewed according to the study plan. 25 patients have been missed and demographic data and outcome data for these patients will be collected and analysed.

## Introduction

It is well known from international studies that emergency laparotomy and other high-risk acute abdominal surgical procedures are associated with a high degree of morbidity and mortality [1–3]. The management of these patients is complex and poses several challenges, not only because of the severe morbidity of the patients but also because the management requires well-functioning, speedy co-operation between different clinics and healthcare professionals.

The incidence of emergency laparotomy and other acute abdominal surgery in Sweden is not known. An estimate from the UK is that 1:1100 [4] in the population requires emergency laparotomy each year. At the departments of anaesthesiology and surgery at Northern Älvsborg County Hospital (NÄL)/NU-Hospital Group, an average of 220 emergency laparotomies are performed annually, i.e. an incidence of approximately 1:1200.

These operations are performed for several reasons, where operations due to ileus, with or without bowel strangulation, operations for acute peritonitis, due to different kinds of stomach or bowel perforation, and re-operations for complications of elective surgery are the most common.

Patients undergoing acute abdominal surgery are all susceptible to negative effects on organ functions in virtually all organ systems due to the underlying condition for which they are undergoing surgery. Sepsis is common and causes or contributes to the impaired organ function [5]. Multi-organ failure (MOF) is sometimes present both pre- and postoperatively.

Several studies have reported an improvement in both mortality and morbidity with standardised management in this group of patients undergoing high-risk acute abdominal surgery [6, 7].

This kind of standardised perioperative protocol has not yet been implemented or studied in Swedish health care. The standard care for these patients in a Swedish setting is a rapid anesthesiological assessment of the patient, preoperative resuscitation—if deemed necessary, followed by surgical intervention. Postoperative care and monitoring are dependent on local routines, facilities, individual assessments of the patients and the patients’ postoperative needs.

## Main text

### Methods

All adult patients at the department of surgery at NÄL requiring acute abdominal surgery are registered and included prospectively in the study. In most cases, the surgical procedure is an emergency laparotomy, but selected cases can start as a laparoscopy.

On inclusion, the patients will be managed according to the standardised protocol. This form will also function as a clinical record form (CRF) for the study. The form will follow the patient during his/her hospital stay and will also be used as a working tool for the medical staff to ensure that the appropriate actions and measurements are implemented as prescribed. An English translated version of the protocol is attached in the Additional file 1 to this article.

#### Perioperative management

Following a decision to operate, the protocol is activated, and the following measures are implemented:

1. Early warning score (EWS; i.e. Heart rate, Blood pressure, Respiratory rate, Saturation, Level of consciousness, and Body temperature) and blood samples: the nurse on the surgical ward measures vital signs (EWS) [6, 8] on the patient. At the same time, extended blood chemical analyses are made. These analyses are haemoglobin concentration, platelet count, white blood count, sodium, potassium, creatinine kinase, CRP, procalcitonin and arterial blood gas [9–12].
2. Physical examination: the responsible surgeon and the anaesthesiologist assess the patient at bedside immediately after notification of surgery [13]. The assessment is supported by the vital signs and the result of blood chemical analysis.
3. Communication: according to the clinical protocol, the anaesthesiologist and the surgeon must communicate how and where preoperative care is best performed. A patient requiring resuscitation before surgery can be moved to intensive care. If the level of care on the ward is adequate, it can be agreed that preoperative care can continue on the surgical ward.
4. Short intervals between the decision to perform surgery and the start of surgery are very important in emergency surgery [6]. Any factor that can delay surgery should be eliminated.
5. Antibiotics: early antibiotic treatment has decreased mortality from sepsis [5]. Patients undergoing acute abdominal surgery have also demonstrated a lower mortality rate with early antibiotic treatment [5, 6].
6. Goal-directed fluid therapy (GDFT): as far as possible, a perioperative assessment of fluid status should be used to optimise the circulation [6, 14, 15].
7. Arterial-line/Invasive blood pressure: this method provides improved control of hemodynamics at the induction of anaesthesia and the ability to follow up the patient’s arterial blood gases peri- and postoperatively [12, 15].
8. Both surgical and anesthesiological assessments of these patients require experience. The aim of the study is to provide the highest possible level of competence in theatre [6].

#### Postoperative management

A high level of postoperative care is important for all the patients in this study; firstly, because the patient’s physiology is often heavily affected and, secondly, because the patient has recently undergone acute abdominal surgery and extensive anesthesiological intervention [16].

Patients that have threatening, or manifest, acute failure in one or more of their vital organ systems are admitted to the ICU after surgery.

The non-ICU postoperative patients in this study will receive upgraded care at recovery. On arrival, an extended blood chemical analysis is performed. After 30 min in recovery, a bedside physical examination is assessed by the responsible anaesthesiologist with the evaluation of labs, fluid therapy and pain therapy. The assessment can be repeated.

When the patient arrives on the surgical ward, an EWS is taken, as well as 2, 4 and 8 h after arrival. The monitoring then continues with an EWS three times daily, until the patient is considered to be stable enough for the EWS to be terminated [8].

#### Inter-professional team

Every month, a team of nurses from the surgical wards, the operating theatre, the postoperative ward and the intensive care unit, a surgeon and an anaesthesiologist review the protocols for the patients included during the last month and check for missed patients.

#### Control group

The cohort of control patients undergoing acute abdominal surgery at NÄL in the years prior to the study will be collected retrospectively. During this period there was no standardisation of the perioperative management of these patients. The management was dependent on the individual responsible surgeon and anaesthesiologist. The therapeutical measures (i.e. start of iv antibiotics, preoperative monitoring, fluid resuscitation, time to surgery etc.) was determined by the level of watchfulness, experience and competence of those in charge/on call. There was no standardisation of start of iv antibiotics, the degree of perioperative monitoring or demand of postoperative assessment by the anaesthesiologist as with the protocol in the intervention group. There was no demand of specific communication between surgeon and anaesthesiologist other than the surgeon making the anaesthesiologist aware of a patient in need for surgery. The measures taken to optimize perioperative care and time to surgery was not standardised and could therefore vary to some degree; both regarding what measures were taken and in what timeframe it was done.

In many cases optimal or near optimal management was sure to be performed but, in some cases, one might suspect—and hypothesise—that measures that would have benefitted the patient was not taken. The control group will therefore by default be comprised of patients managed within a span of medical measures and time, and the hypothesis of the study is that a standardised protocol-based management of patients with pre-defined measures is superior to such management.

For the control group medical data—including time from decision to surgery, start of iv antibiotics, degree of perioperative monitoring, formal competence of those involved etc.—and outcome will be collected from the patients’ medical records.

#### Study and publication plan

**Fig. 1:**
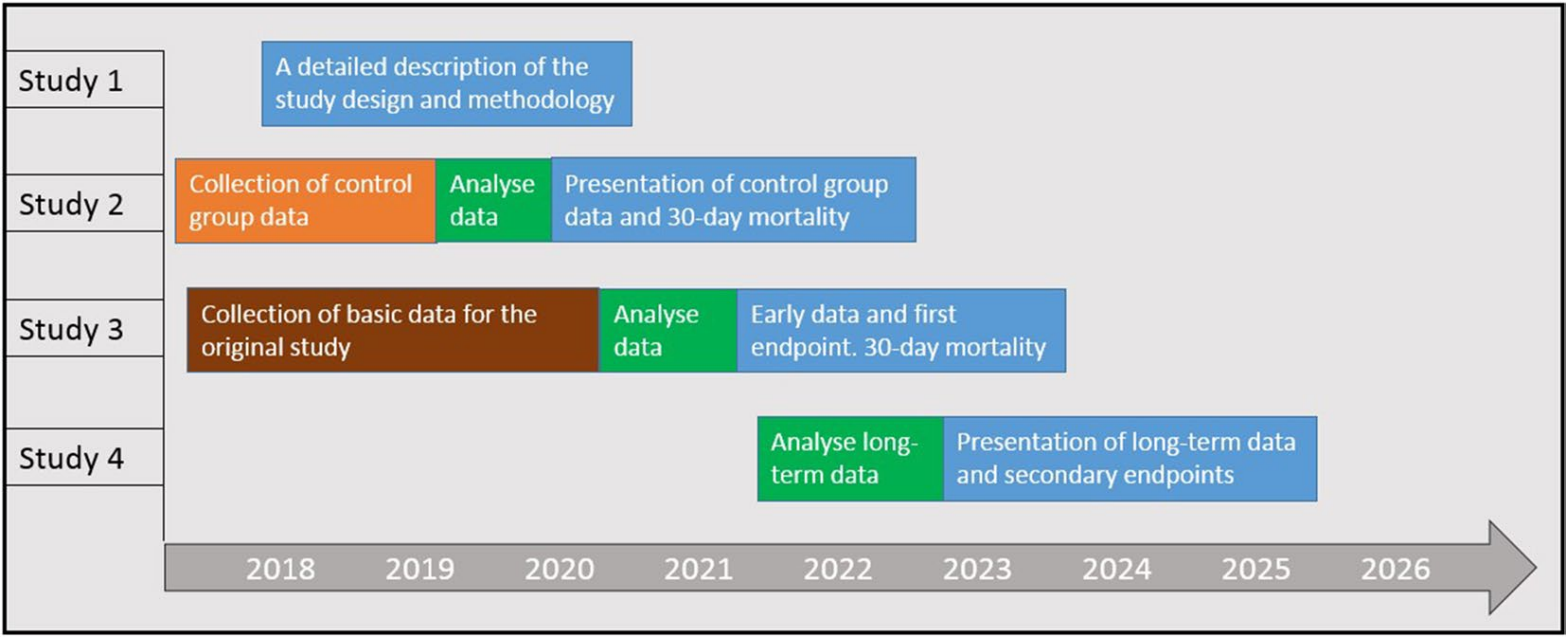
The overall plan for the study implementation in terms of data collection, analysis and presentation of results

The plan for data collection and publication (Fig. 1):

### Results

There are no results from the study so far. Inclusion of the intervention group is ongoing: In the first 22 months of the study, 404 patients have been included and protocols have been followed and reviewed according to the study plan. 25 patients have been missed, but demographic data and outcome data for these patients will be collected and analysed in order to detect selection bias and to have complete records for all eligible patients. Data on the control group are being collected according to the study plan and simple univariate analyses of these data will be performed within short.

### Discussion

It is well known that acute abdominal surgery is associated with high morbidity and mortality [1–3].

The primary aim of this study is to investigate whether a standardised protocol is able to improve the outcome following acute abdominal surgery. As far as we know, this is the first time this has been done in a Swedish context.

The study is designed as a single-centre study, this can be regarded as a possible weakness, as the external validity could decrease with just one centre including patients. We believe, however, that it will be possible to generalise the results of our study based on the size of the study population, the fact that all the patients requiring acute abdominal surgery are included and the measures medical strategies that are used are no different from the standard in modern surgical and anesthesiological care. Other limitations of the single-centre design are that it takes more time to collect a sufficiently large study group. This allows room for standard treatment for a specific pathology to change over the years and the result can be affected.

We regard it an advantage to have local control of the registration of included patients and this facilitates the ability to continuously follow up on patient inclusion. Another strength of the study is that the cohort of controls is collected from surgery performed at the same centre during the years prior to the study, which enables the control group and study group to be as homogeneous and comparable as possible.

If the study were conducted as a multicentre study, there could also be an increased risk of loss of compliance with the protocol and the registration of data, due to the added complexity in the study organisation.

One answer to the limitations mentioned above would be randomisation, an RCT. However, there are difficulties involved in randomising this study. There are strong indications that the combination of measurements and actions we standardise are beneficial to the patients, even if the extent is not fully known and we do not suspect that the standardisation would be harmful to the patients.

With this in mind, it would be ethically doubtful to randomise patients to different treatment strategies. In a randomised study, there is also a risk of intervention transferral, i.e. in the treatment of patients in the control group, there would be a risk of clinicians still following the standardised protocol, thus diluting the differences between the intervention and the control group.

Efforts have been made to inform all the personnel about the standardised care of these patients and information is given continuously. The large number of healthcare workers involved could be regarded as a factor that increases the external validity of the study, but there is always an inherent weakness with so many involved parties delivering data in a study.

We think that the presence of an inter-professional team that regularly reviews each case and double-checks that protocols are correctly registered improves the reliability of data. All departments and professions are represented in the inter-professional team. The team continuously gives feedback on areas that might need improvement. This kind of multi-professional collaboration with many different healthcare professionals may also foster implementation and adherence to the standardised management [17].

In conclusion, this study is being performed with the aim of improving the outcome for a large group of patients running a high risk of severe postoperative morbidity and mortality. The hypothesis that a standardised protocol for the perioperative care of these patients improves outcome has a solid basis in the literature, but it has not yet been studied or implemented in a Swedish setting.

## Limitations

- The single-centre design gives a decrease in the external validity.
- We do not randomize patients in the study.
- The retrospective control group extends the study period and standard treatment for a specific pathology may change over the years.
- Some data are missing for the control group because it is collected retrospectively.

## Supplementary information

**Additional file 1.** CRF English version.

## Data Availability

Data sharing is not applicable to this article as no datasets were generated or analysed so far. This article is primarily a method description, data from the study is not presented except for the number of included and missed patients we have in the study group up to this point. When the study is completed and the data is analysed, the result will be published and together with it the dataset will be made available.

## Abbreviations

MOF: Multi organ failure
NÄL: Northern Älvsborg County Hospital/NU-Hospital Group
CRF: Clinical record form
EWS: Early warning score
GDFT: Goal-directed fluid therapy
ICU: Intensive care unit
RCT: Randomised Controlled Trial
