# Supplementary material for: SMASH standardised perioperative management of patients operated with acute abdominal surgery in a high-risk setting
Source: BMC Res Notes. 2020 Mar 31;13:193. doi: 10.1186/s13104-020-05030-4 (PMC7110666; doi:10.1186/s13104-020-05030-4)
Supplement: Supplementary file 1 — Additional file 1. CRF English version. [file 13104_2020_5030_MOESM1_ESM.pdf]

VÄSTRA GÖTALANDSREGIONEN
NU-SJUKVÅRDEN

Emergency Laparotomy

This CRF follows the patient from the decision of surgery and until the patient being discharged from hospital.

When patient is discharged, send form to:
Contact: Dr. Mattias Prytz, Consultant
mattias.prytz@vgregion.se tel: +46104353523

Surgical code: **JAH00 Emergency Laparotomy**. Add code **JAH01** if laparoscopy.

Priority:
- ☐ emergency
- ☐ 2 hours
- ☐ 6 hours

PATIENT DATA

Preoperative- Surgeon liability

Surgery notification: date ________time____

- ☐ Latest **surgical** bedside assessment before notification. Time:_______
- ☐ Reoperation after elective surgery
- ☐ Surgery will initiate as laparoscopy

The following are carried out **immediately** after a decision to operate has been taken.

- ☐ **Antibiotics** given, time:_______
  Septic patient- 2:nd dose after 4h.
- ☐ **EWS-early warning score**
  Register in med journal. score: ____
- ☐ **Arterial Blood Gas** with lactate
- ☐ **extended blood chemical analyses**.
  Hb, platelet count, white blood count, sodium, potassium, creatinine, CRP, procalcitonin
- ☐ **Urinary catheter**
- ☐ **Nasogastric tube**
- ☐ **Planning** of the continuing pre-operative care in consultation with the responsible anesthetist.
  (**All** factors that can delay surgery must be eliminated, i.e. shower.)

The following is done **promptly** and must be completed before surgery.
Decide on care restrictions. Specify below if restrictions apply.
- ☐ yes
- ☐ no

- ☐ **Relatives contacted**. Reason if not contact______________________
  ____

Comorbidity

- ☐ COPD
- ☐ Ischemic heart disease
- ☐ Heart failure
- ☐ Diabetes
- ☐ Kidney failure
- ☐ Obesity
- ☐ Smoker
- ☐ None of the above

Peroperative- Anesthetist liability

- ☐ **Anesthestist** preop bedside assessment
  Time _____ ASA-classification:_____

Ready for theater:_____ Anesthesia start:_____

Surgery start:_______surgery finish:_______

- ☐ **Thoracic Epiduaral**
  If the Epidural is not perfomed, please state the reason below:____________
- ☐ **A-line**
- ☐ **Noradrenaline**
- ☐ **RSI**
  Drug for induction.
  - ☐ Ketamin
  - ☐ Propofol
- ☐ **Central venous catheter**
  Should especially be considered when there is a need for post-care at ICU.

Goal directed fluid therapy (GDFT):
- ☐ **CardioQ**
- ☐ **Lidco-Rapid**
- ☐ **PPV**
- ☐ **other** ________________________

Decision on post-op level of care after consultation between surgeon and anesthetist.
- ☐ Post-op **recovery**
- ☐ Post-op **Intensive care unit**
  Reasons for postop-ICU
  ____
  ____

Anesthesiological complication with postoperative significance
- ☐ No complication
- ☐ Aspiration
- ☐ Other__________________________
  ____

Per-operativ
Competence of Surgeon
- ☐ Resident
- ☐ Specialist
- ☐ Consultant

Per-operativ
competence of Anesthetist
- ☐ Resident
- ☐ Specialist
- ☐ Consultant

Post-op-recovery Nurse liability

Recovery start: date_______ time_______

Recovery finish: date_______ time_______

The following is performed at recovery before round with anesthesiologist.

- ☐ **monitoring** standard
- ☐ **Temperature _______ degrees.**
- ☐ **Chemical blood analyse,** ICU-standard
- ☐ **Arterial Blood Gas**
- ☐ **Active temperature control**
- ☐ **Visual Analog Scale** on arrival:____
- ☐ **EWS-early warning score.** Register in medical journal. score: ______
- ☐ **Visual Analog Scale** on discharge:_____

Post-op- Anesthetist liability

After approximately **30 minutes** at recovery.

Assessment bedside by anesthesiologist:
Review of treatment goals. Time:________
Patient should be assessed every two hours unless vital parameters show a completely stable patient.
Goal directed fluid therapy post-op:
- ☐ Lidco-Rapid
- ☐ CardioQ
- ☐ other__________________
- ☐ none

Evaluation of **paintreatment** post-op:
- ☐ Epidural
- ☐ Patient Controlled Analgesia
- ☐ TAP-Block
- ☐ PainBuster
- ☐ Other___________________.

Care continues at:
- ☐ Surgical ward
- ☐ ICU
- ☐ Other________________

Post-op surgical ward: Nurse liability

Arrival to ward: date_____ Time_____

- ☐ EWS on arrival. score:____
- ☐ EWS 2 h after arrival. score:____
- ☐ EWS 4 h after arrival. score:____
- ☐ EWS 8 h after arrival. score:____

EWS every 8 hours as long as the responsible surgeon sees the need.
Take **immediate** contact with a surgeon in case of clinical deterioration.
EWS-evaluation terminates at. date:______

Pathological findings and surgical procedure: ____________________________________-
____

Post-op complication Surgeon liability (Not to be filled in to early)

A postoperative complication has arisen that….: Date when reviewed:_____________
- ☐ 1) is pharmacological treated with: Antiemetics, antipyretics, analgesics, diuretics and/or electrolytes
- ☐ 2) is treated with pharmacotherapy not mentioned above (eg blood transfusion or TPN).
- ☐ 3a) requires surgical, endoscopic or radiological intervention.
- ☐ 3b) requires surgical, endoscopic or radiological intervention in general anesthesia.
- ☐ 4a) requires ICU care due to single organ failure.
- ☐ 4b) Requires ICU care due to multiorgan failure.
- ☐ 5) lead to patient loss of life.

Reviewed by:
